# Molecular Breeding of a Fungus Producing a Precursor Diterpene Suitable for Semi-Synthesis by Dissection of the Biosynthetic Machinery

**DOI:** 10.1371/journal.pone.0042090

**Published:** 2012-08-01

**Authors:** Motoyoshi Noike, Yusuke Ono, Yuji Araki, Ryo Tanio, Yusuke Higuchi, Hajime Nitta, Yoshimitsu Hamano, Tomonobu Toyomasu, Takeshi Sassa, Nobuo Kato, Tohru Dairi

**Affiliations:** 1 Graduate School of Engineering, Hokkaido University, Hokkaido, Japan; 2 The institute of Scientific and Industrial Research, Osaka University, Osaka, Japan; 3 Department of Bioscience, Fukui Prefectural University, Fukui, Japan; 4 Department of Bioresource Engineering, Yamagata University, Yamagata, Japan; University of New South Wales, Australia

## Abstract

Many clinically useful pharmaceuticals are semi-synthesized from natural products produced by actinobacteria and fungi. The synthetic protocols usually contain many complicated reaction steps and thereby result in low yields and high costs. It is therefore important to breed microorganisms that produce a compound most suitable for chemical synthesis. For a long time, desirable mutants have been obtained by random mutagenesis and mass screening. However, these mutants sometimes show unfavorable phenotypes such as low viability and low productivity of the desired compound. Fusicoccin (FC) A is a diterpene glucoside produced by the fungus *Phomopsis amygdali*. Both FC and the structurally-related cotylenin A (CN) have phytohormone-like activity. However, only CN exhibits anti-cancer activity. Since the CN producer lost its ability to proliferate during preservation, a study on the relationship between structure and activity was carried out, and elimination of the hydroxyl group at position 12 of FC was essential to mimic the CN-like activity. Based on detailed dissection of the biosynthetic machinery, we constructed a mutant producing a compound without a hydroxyl group at position 12 by gene-disruption. The mutant produced this compound as a sole metabolite, which can be easily and efficiently converted into an anti-cancer drug, and its productivity was equivalent to the sum of FC-related compounds produced by the parental strain. Our strategy would be applicable to development of pharmaceuticals that are semi-synthesized from fungal metabolites.

## Introduction

**Figure 1:**
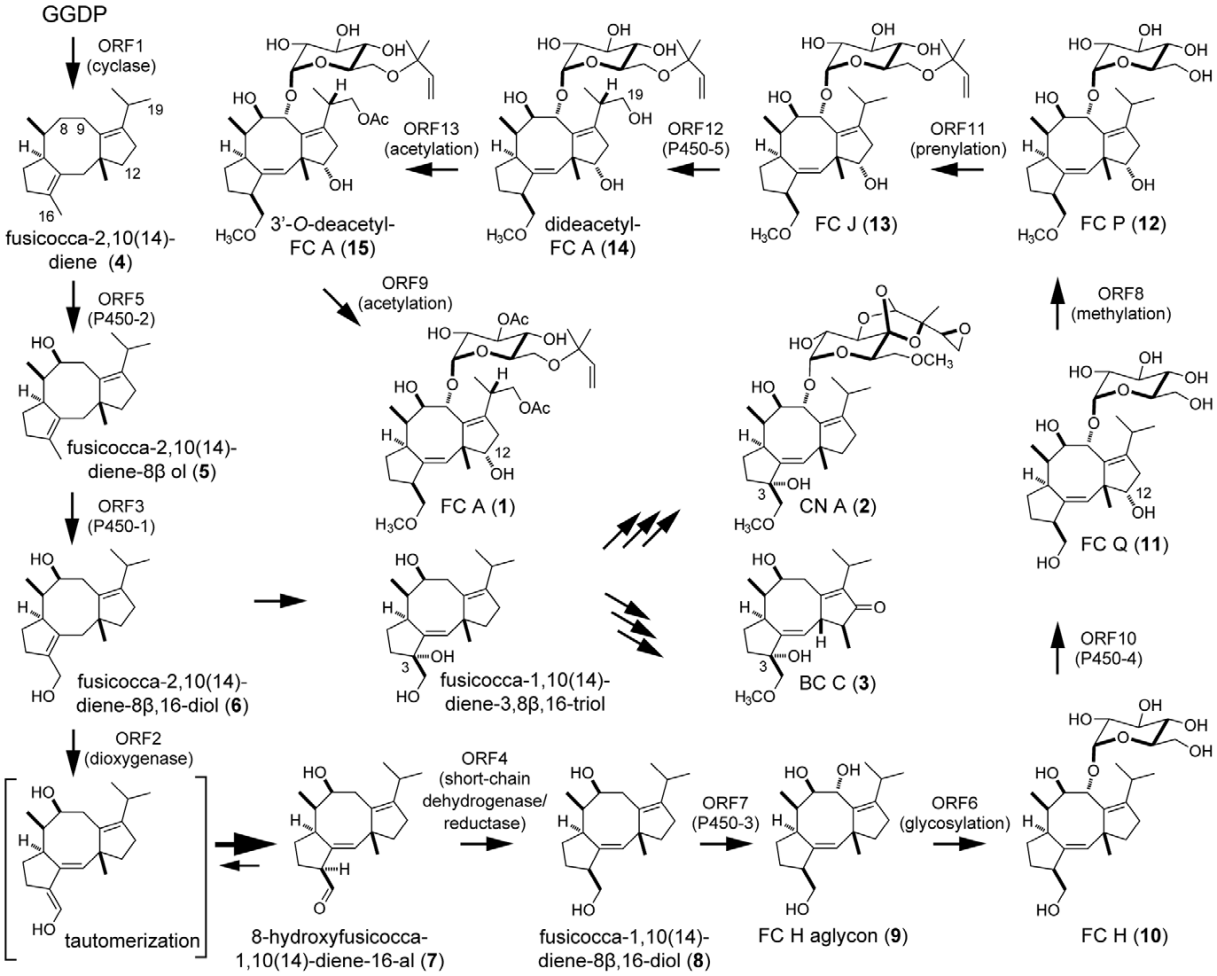
Proposed biosynthetic pathways for FC A, CN A, and BC C.

For screening of candidate compounds useful for pharmaceutical drugs, *in vitro* assay methods are often employed. The recent development of robotic high throughput screening of large chemical libraries enables us to screen many compounds at once. When a compound is selected by such screening, synthesis of its derivatives would be relatively easy because the original library was chemically constructed. On the other hand, screenings from natural products still occupy an important position because many clinically useful pharmaceuticals originate from natural products. However, most natural products are chemically modified for clinical use to decrease toxicity and increase solubility. The synthetic protocols for these chemical modifications sometimes contain many complicated reactions and thereby result in low yields and high costs. To overcome this problem, a strategy of mutagenesis of the producers with mutagens such as *N*-Methyl-*N*'-nitro-*N*-nitrosoguanidine has been employed, expecting that a more suitable compound for chemical synthesis might be produced rather than a final product. However, this type of trial was usually unsuccessful because the mutants showed low viability and productivity. Therefore, it is essential to dissect the biosynthetic machinery of the compound and to make use of this knowledge for breeding of the producer. In this study, we applied this new strategy to a fungus producing FC A (**1**) (Fig. 1) [1], a diterpene glucoside.

Both **1** and the structurally-related cotylenin A (**2**) (Fig. 1) [2], [3] are diterpene glucosides produced by fungi that show phytohormone-like activity [4]. Recently, only **2** was shown to induce the differentiation of human myeloid leukemia cells and trials to apply **2**-derivatives to anti-cancer drugs are in progress [5], [6], [7], [8], [9], [10]. However, the **2** producer, which was isolated more than 40 years ago and classified as *Cladosporium* sp. by classical methods such as morphology [5], had lost its ability to proliferate because of long preservation on a slant. We tried to isolate a producer of **2** or **2**-related compounds by mass screening but could isolate only producers of **1** and brassicicene (BC) C (**3**) (Fig. 1) [11], a compound structurally-related to **1**. This resulted in an inability to prepare a large amount of **2** for further clinical application. A study on the relationship between structure and activity was then carried out with **1** and elimination of the hydroxyl group at position 12 was found to be essential for **2-**like activity. However, the sole existing synthetic protocol, which is technically applicable to industry, to remove the 12-hydroxyl group is inefficient and involves many reaction steps from FC J (**13**) (Fig. 1) [12], a minor biosynthetic intermediate slightly accumulated in culture broth of the **1** producer (Fig. 1). We recently developed a more efficient synthetic route from FC H (**10**) (Fig. 1) [13], which is another intermediate lacking a hydroxyl group at position 12 and accumulated less than **13** in the culture [10].

**Figure 2:**
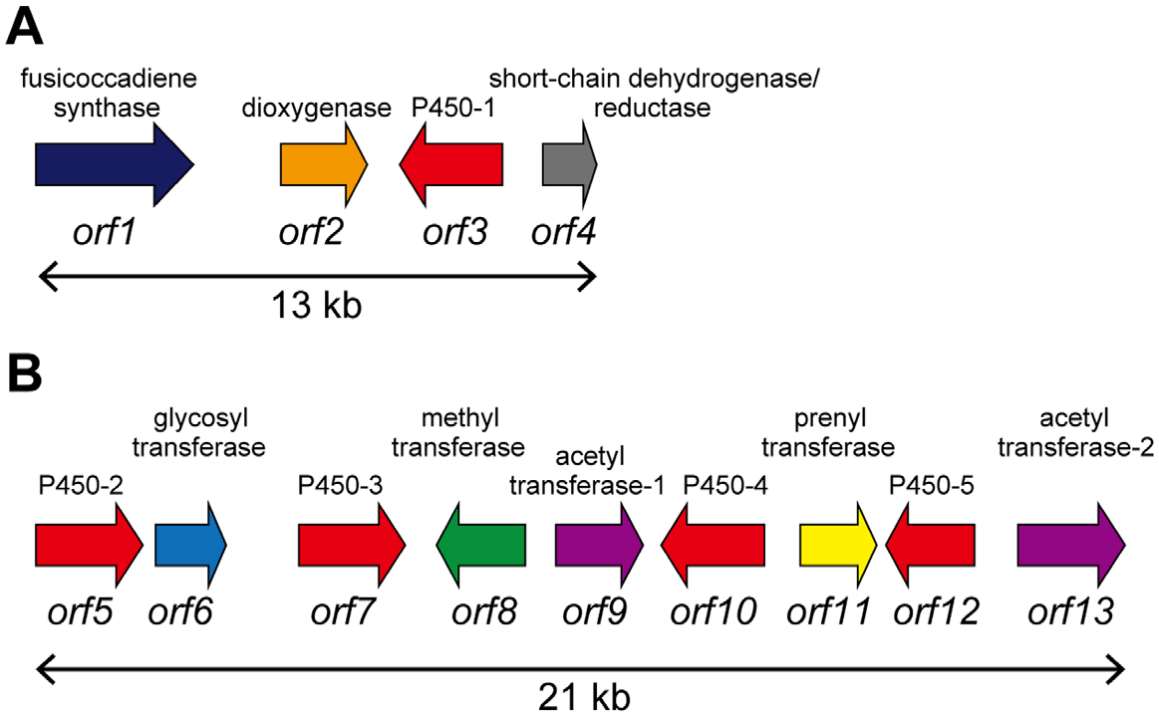
Fusicoccin A biosynthetic gene clusters cloned in our previous (A) and this study (B), respectively.

To estimate whether breeding of a strain that produces **10** as a main product was possible, we started to dissect its biosynthetic machinery. We previously identified a gene encoding PaFS (Orf1) from *Phomopsis amygdali*, which is a unique chimeric enzyme possessing both a geranylgeranyl diphosphate synthase domain and a diterpene cyclase domain [14]. In addition, three genes, α-ketoglutarate dependent dioxygenase (*Orf2*), cytochrome P450 (*Orf3*, accession number; AB68627), and short-chain dehydrogenase/reductase (*Orf4*) were clustered near the *Orf1* gene (Fig. 2A). Orf2 was confirmed to catalyze the 16-oxydation of fusicocca-2,10(14)-diene-8β,16-diol (**6**) to yield 8β-hydroxyfusicocca-1,10(14)-dien-16-al (**7**), followed by reduction of the aldehyde to yield fusicocca-1,10(14)-diene-8β,16-diol (**8**) by Orf4 (Fig. 1) [15]. However, no genes related to **1** biosynthesis were identified in upstream and downstream regions by genome walking (about 50 kbp each). By draft genome sequencing, we then identified a gene (*papt*) at another locus that encodes a prenyltransferase (Orf11) catalyzing a reverse transfer of dimethylallyl diphosphate to the 6′-hydroxy group of glucose moiety of FC P (**12**) (Fig. 1) [16], [17]. Since our goal is the identification of other genes involved in **1** biosynthesis, we did not determine the complete genome sequences and were not able to estimate the distance between the two clusters. However, the remaining genes were identified in the flanking region of *papt* in this study. We performed detailed analysis of the enzymes catalyzing the reactions after the formation of **10**. Kinetic parameters of the enzymes suggested that the enzymes could accept only intrinsic substrates and not **10**. We therefore identified and disrupted a gene responsible for hydroxylation at the 12-position, which would be a biosynthetic reaction just after **10** formation. The disruptant produced **10** as a main metabolite as expected, which can be easily and efficiently converted into the anti-cancer drug, and its productivity was equivalent to the sum of FC-related compounds produced by the parental strain.

## Results

### Identification and Characterization of Enzymes Responsible for Late Biosynthetic Steps of 1

To examine whether the remaining **1** biosynthetic genes were clustered with the *papt* gene, we searched for flanking contigs by gene walking. Consequently, we identified another gene cluster (21 kbp) containing nine genes; four cytochrome P450s (Orf5; AB686271, Orf7; AB686273, Orf10; AB686276 and Orf13; AB686278), two acetyltransferases (Orf9; AB686275 and Orf12; AB686277), a methyltransferase (Orf8; AB686274), a glycosyltransferase (Orf6; AB686272), and a prenyltransferase (Orf11) (Fig. 2B), suggesting that the **1** biosynthetic genes are scattered at two different loci; one contained four genes and the other nine.

Our goal was the breeding of a mutant accumulating **10**. Based on the putative **1** biosynthetic pathway, which was estimated by analyses of intermediate compounds accumulated in the culture broth of the **1** producer [16], a 12-hydroxylation gene-disruptant was expected to accumulate **10** (Fig. 1). However, the **1** biosynthetic machinery might skip this 12-hydroxylation reaction since the “metabolic grid pathway” is well known in the biosynthesis of secondary metabolites, and accumulation of several 12-dehydroxy derivatives of **1** was also probable. Among such compounds, however, no accumulation of 12-dehydroxy FC J and 12-dehydroxy- 3′-*O*-deacetyl FC A (**15**) (Fig. 1) was suggested by the following observation: the wild type strain accumulates **15** and **13** in addition to **1** in almost the same amounts in a typical fermentation, suggesting that the catalytic activities of the two enzymes utilizing **15** (3′-*O*-acetylation) and **13** (19-hydroxylation) as substrates are very low even with the intrinsic substrates and that **10**, the intermediate in the early biosynthetic step, would not be accepted as a substrate. Therefore, we investigated the detailed properties of a methylation enzyme and a prenylation enzyme, both of which participate in reactions after the formation of **10**. To make a precise interpretation, enzymatic properties of acetylation and glycosylation enzymes were also studied and compared to those of the methylation and prenylation enzymes.

#### (i) Methylation

**Figure 3:**
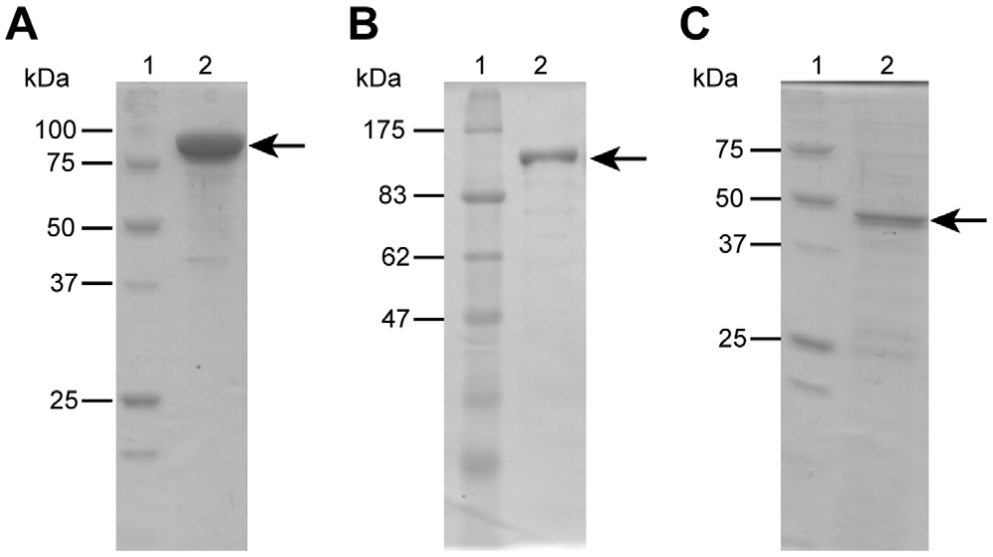
SDS-PAGE analysis of the purified enzymes. (A) molecular mass markers (lane 1) and purified methyltransferase (lane 2). (B) molecular mass markers (lane 1) and purified acetyltransferase (lane 2). (C) molecular mass markers (lane 1) and purified glycosyltransferase (lane 2).

The reaction followed by the 12-hydroxylation would be a methylation of the hydroxyl group at position 16 of FC Q (**11**) (Fig. 1) [16] by Orf8, which has high similarity to many methyltransferases that use *S*-adenosyl-L-methionine (SAM) as a methyl donor. A recombinant Orf8, which was shown to be 89 kDa by SDS-PAGE (Fig. 3), was incubated with **10** and FC H aglycon (**9**) (Fig. 1) [13] since we did not have sufficient **11**, a plausible intrinsic substrate. Although both compounds were accepted as substrates, the amount of product formed from **9** was trace (Fig. S1A) and the structure of the product from **10** was confirmed to be 16-*O*-methyl-**10** by LC/MS analysis (Fig. S1B). When a mixture of **10** and 3-*epi*-**10** [16] (3.7∶1) were used as substrates, only **10** was converted into methylated product (Fig. S1A), suggesting that the enzyme recognizes a stereochemistry at position 3 of the substrate. Kinetic studies were then performed with **10** as the substrate. The optimum pH and temperature were 9.0 and 35°C, respectively. The *Km* values were calculated as 17±1.8 µM for **10** and 56±6.0 µM for SAM with a *kcat* of 0.043±0.0016 S^−1^. The *kcat*/*Km* value was very small compared to other SAM-dependent methyltransferases, suggesting that **11** would be an intrinsic intermediate as previously reported.

#### (ii) Prenylation

We previously confirmed that the Orf11 product catalyzed the prenylation of **12** and **10** [17]. We also showed that the *kcat/Km* value for **12** was 50 times higher than for **10**, suggesting that **12** is an intrinsic substrate for **13** formation *in vivo*.

#### (iii) Acetylations

Orf12 and Orf9 had significant similarity to a cytosolic acetyltransferase and another type of membrane bound acetyltransferase, respectively. Considering the structure of **1**, the hydroxyl groups at the 3′-position and 19-position of dideacetyl-FC A (**14**) (Fig. 1) [18] should be acetylated by these enzymes. A recombinant ORF12, shown to be 103 kDa by SDS-PAGE (Fig. 3), was incubated with **14** and acetyl-CoA and the reaction product was analyzed by HPLC. A single product was detected and its structure was confirmed to be 3′-*O*-deacetyl-FC A (**15**) [19] by LC/MS analysis (Fig. S2). The optimum pH and temperature were 8.5 and 35°C, respectively. The *Km* values were calculated as 170±4.0 µM for **14** and 63±6.6 µM for acetyl-CoA with a very low *kcat* value, 3.5×10^−3^±0.17×10^−3^ S^−1^, which would result in significant accumulation of **14** in the culture broth of the **1** producer. We also tried to prepare a recombinant Orf9, a probable membrane bound acetyltransferase, in *E. coli*, *Saccharomyces cerevisiae, Pichia pastoris* and *Aspergillus oryzae*. However, no expression of recombinants was observed. Since the enzyme introducing a hydroxyl group at the 19-position probably could not accept **10** as a substrate as mentioned above and the 19-hydroxylation should precede the acetylation, we paid no more attention to this acetylation reaction from the viewpoint of **10** biosynthesis.

#### (iv) Glycosylation

Since glycosylation of **9** forms **10**, the enzymatic properties of the glycosylation enzyme (Orf6) were also investigated. A recombinant enzyme, shown to be 46 kDa by SDS-PAGE (Fig. 3), was prepared and incubated with **9** in the presence of UDP-glucose as a glucose donor. A specific peak was detected by HPLC analysis and confirmed to be **10** by LC/MS analysis (Fig. S3). The optimum pH and temperature were 5.5 and 35°C, respectively. The *Km* values were calculated as 44±8.1 µM for **9** and 520±46 µM for UDP-glucose with a *kcat* of 0.40±0.044 S^−1^, which is more than ten times higher than those of the methylation (Orf8), prenylation (Orf11), and acetylation (Orf12) enzymes, suggesting that the glycosylation is not a rate-limiting step.

Based on the low catalytic activities of methyltransferase and prenyltransferase calculated with **10** as the substrate and the metabolite profile of the wild strain, we expected that 12-hydroxylation gene-disruptant would accumulate **10** as a main product.

### Identification of the Gene Responsible for 12-hydroxylation

Targeted gene disruption is difficult with fungi because a random integration of exogenous DNA into genomic DNA is usually more likely than a homologous recombination. We then examined the substrate specificities of five P450s (Orf3, 5, 7, 10, and 13), one of which should catalyze 12-hydroxylation, by *in vitro* experiments.

#### (i) Hydroxylation at positions 8 and 16

P450-2 (Orf5) and P450-1 (Orf3) has significant similarities to BC-Orf1 and BC-Orf7, respectively, which were identified in *Alternaria brassicicola*, a producer of **3** [20]. We previously confirmed that the former enzyme catalyzed the formation of fusicocca-2,10(14)-diene-8β-ol (**5**) by hydroxylation of fusicocca-2,10(14)-diene (**4**), followed by 16-hydroxylation to form fusicocca-2,10(14)-diene-8β,16-diol (**6**) by the latter enzyme (Fig. 1) [21]. Since **6** would be the same intermediate between **3** and **1** biosynthesis, P450-2 (Orf5) and P450-1 (Orf3) perhaps have the same catalytic activities of those of BC-Orf1 and BC-Orf7. We therefore examined the function of P450-2 (Orf5) by the same method. *S. cerevisiae* transformants carrying fusicocca-2,10(14)-diene synthase, cytochrome P450 reductase (AB686279), and P450-2 genes were cultivated and their product was analyzed by GC/MS analysis and confirmed to be **5** (Fig. S4), suggesting that P450-2 (Orf5) and P450-1 (Orf3) have 8- and 16-hydroxylation activities.

#### (ii) Hydroxylation at position 9

We previously confirmed that **6** was converted to **8** by successive reactions by the dioxygenase (Orf2) and the short chain dehydrogenase/reductase (Orf4) [15]. Therefore, the next intermediate is **9**, which would be formed by Orf7, Orf10, or Orf13. To narrow this down, we expressed each of the P450 genes in microsomes of *S. cerevisiae* together with the cytochrome P450 reductase gene and used the isolated microsomes as enzyme sources with **8** as the substrate. We detected a specific product by HPLC analysis only when microsomes expressing P450-3 (Orf7) were used as the catalyst. The compound was identified to be **9** by LC/MS analysis (Fig. S5), suggesting that P450-3 (Orf7) is the 9-hydroxylation enzyme and that P450-4 (Orf10) and P450-5 (Orf13) should be involved in hydroxylation at 12- and 19-positions. We then examined this possibility by the same method, but no products were formed in either case.

### Disruption of *orf10* and *orf13*

**Figure 4:**
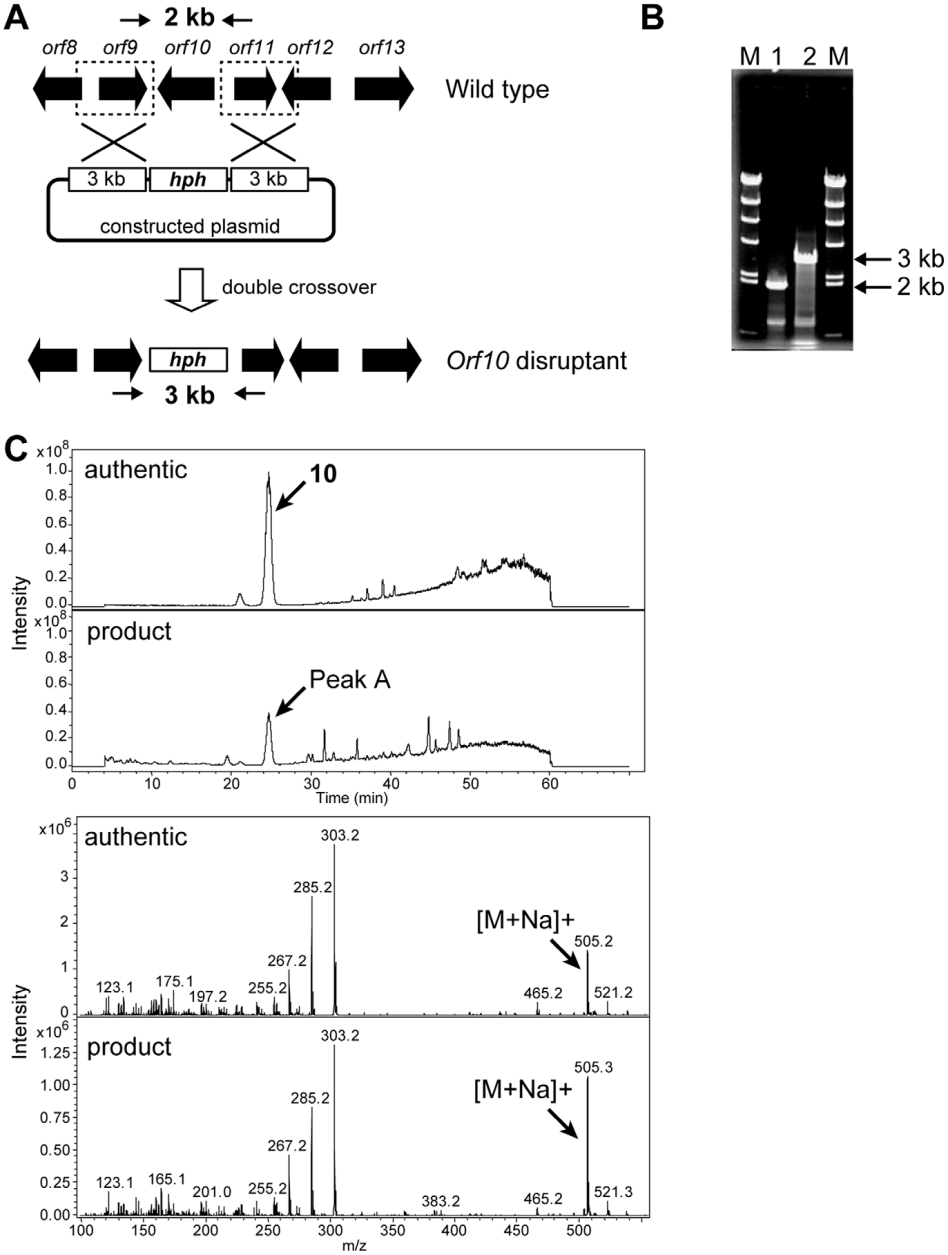
Construction of the *orf10* disruptant and LC/MS analyses of the product accumulated in a culture broth of the *orf10* disruptants. (A) A strategy for disruption of the *orf10* by a double crossover is schematically shown. Arrows indicate the primers (Table S1) used in PCR analysis, which correspond to the upstream and downstream of the *orf10*. (B) Disruption was confirmed by agarose gel electrophoresis of the PCR-amplified fragment. (C) The product accumulated in a culture broth of the *orf10* disruptant (peak A) was confirmed to be **10** by LC/MS analysis.

**Figure 5:**
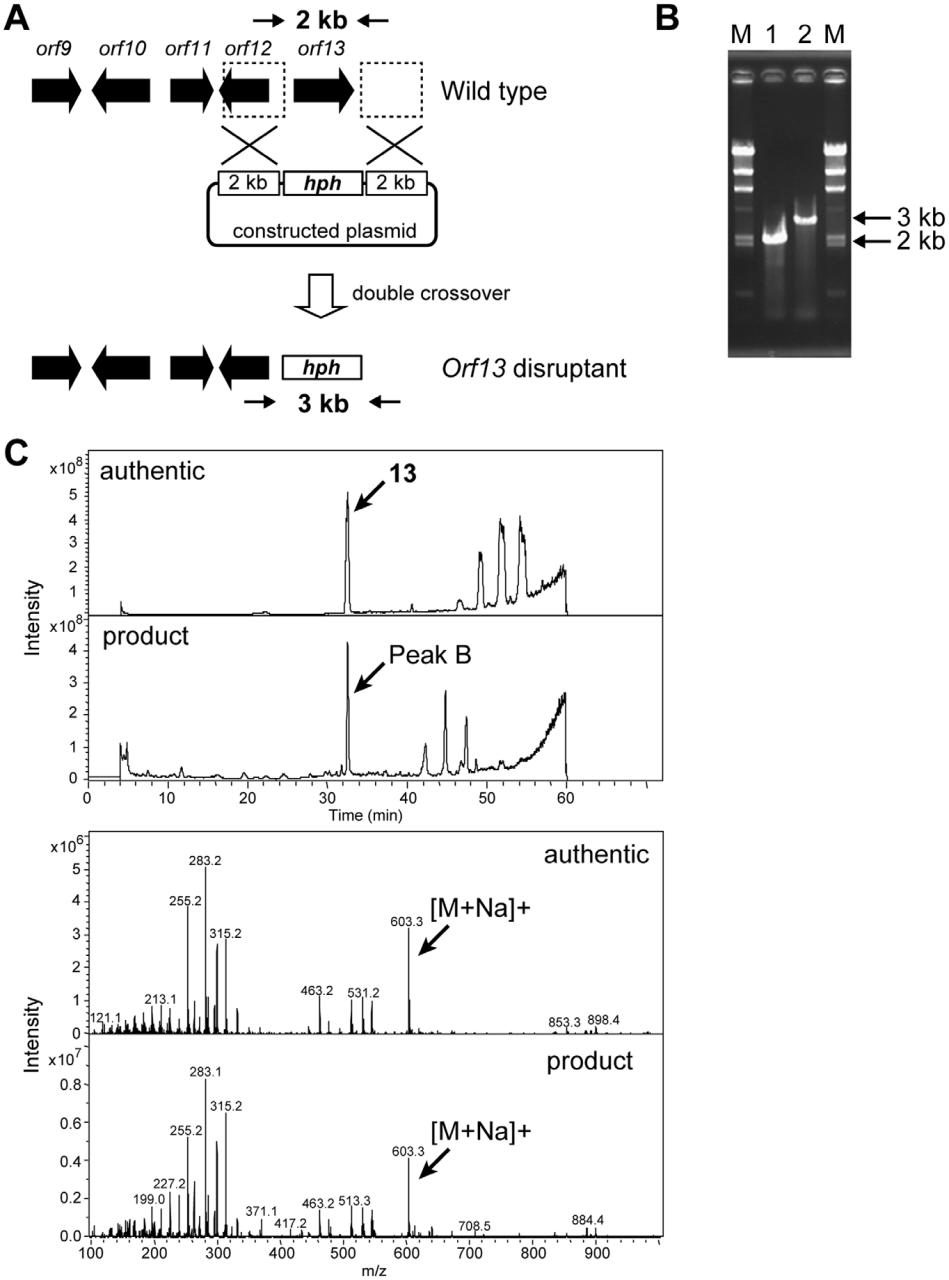
Construction of the *orf13* disruptant and LC/MS analyses of the product accumulated in a culture broth of the *orf13* disruptants. (A) A strategy for disruption of the *orf13* by a double crossover is schematically shown. Arrows indicate the primers (Table S1) used in PCR analysis, which correspond to the upstream and downstream of the *orf13*. (B) Disruption was confirmed by agarose gel electrophoresis of the PCR-amplified fragment. (C) The product accumulated in a culture broth of the *orf10* disruptant (peak B) was confirmed to be **13** by LC/MS analysis.

**Figure 6:**
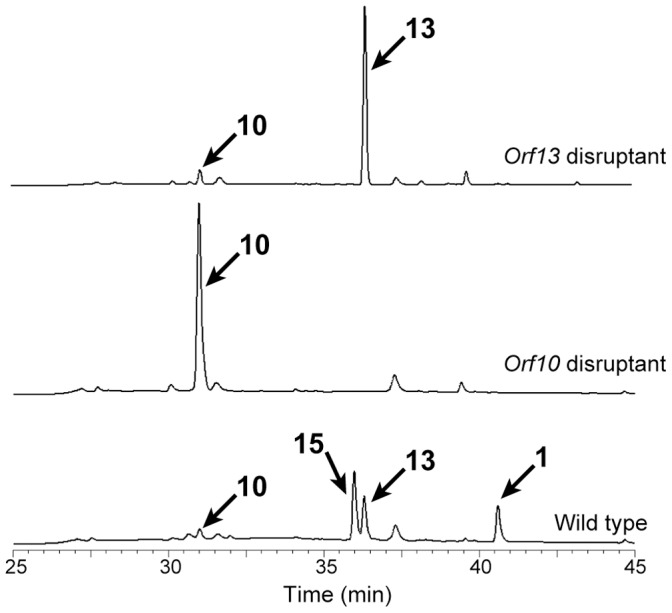
HPLC traces of the broths of the orf13 disruptant (upper), the orf10 disruptant (middle), and the parental strain (lower).

We therefore disrupted the P450-4 (*orf10*) and P450-5 (*orf13*) genes by homologous recombination, expecting that one of the disruptants would produce **10**. A plasmid, in which *orf10* (Fig. 4A) or *orf13* (Fig. 5A) was replaced with the hygromycin resistance gene, was constructed and introduced into the **1** producer. Hygromycin-resistant transformants were selected and the homologous recombination was examined by PCR with appropriate primers and genomic DNA of transformants as a template (Fig. 4C and 5C). In both cases, the intended disruptants emerged at a frequency of less than 0.1% of transformants. Next, the product accumulated in the culture broth of the disruptants was examined. The P450-4 (*orf10*)-disruptant and the P450-5 (*orf13*)-disruptant produced **10** and **13**, respectively, confirmed by LC/MS (Fig. 4B and 5B), as the main product as expected and their productivities were equivalent to the sum of **1**-related compounds produced by the parental strain (Fig. 6).

## Discussion

To date, production of many useful compounds by microorganisms has been achieved. High-titer production of amino acids, nucleic acids, and antibiotics, producers of which were mostly bred by classical mutagenesis and mass screenings, are representative. Recently, synthetic biology and metabolic engineering has enabled successful production of specific, valuable compounds, including those of eukaryotic origin, with *Escherichia coli* and yeast as hosts [22], [23], although expression optimization for each of the genes/enzymes introduced into the hosts is indispensable and greater titer is essential for industrial application. Besides these processes, in this study, we demonstrated an additional process: production of an intermediate compound suitable for semi-synthesis by a mutant constructed by disruption of a specific gene by homologous recombination. It is worth mentioning that the disruptants produced the intermediates as sole products and that their productivities were the identical to the end product produced by the parental strain (0.6 g/L broth). From the viewpoint of industrial application, **10** accumulated in the culture broth of the P450-4 (*orf10*)-disruptant easily crystallized after filtration and successive ethyl acetate extraction, which enabled its direct use for semi-synthesis at low cost.

There are few reports on the productivities of intermediate compounds produced by engineered strains bred by classical mutagenesis and/or metabolic engineering. Moreover, to the best of our knowledge, there are no reports on detailed enzymatic properties of a series of biosynthetic enzymes responsible for a secondary metabolite. In this study, we demonstrated the strict substrate specificities of **1** biosynthetic enzymes. This would be the reason why the disruptant accumulated the specific intermediate as the sole product without utilizing the “metabolic grid pathway”, which is sometimes observed in the biosynthesis of secondary metabolites. In gibberellin biosynthesis, high accumulation of *ent*-kaurene, *ent*-kaurenoic acid, and gibberellin 14 by *Fusarium fujikuroi* mutants, in which the P450-4, P450-1, and P450-2 genes responsible for gibberellin biosynthesis were disrupted by homologous recombination, were also reported [24]. Therefore, fungi would generally have the potential to accumulate an intermediate compound, at least for isoprenoid secondary metabolites.

We have been studying biosynthetic machinery of actinobacteria such as *Streptomyces* strains [25], [26], [27], [28], counterparts of natural product producers, and have tried to breed isoprenoid producers that accumulate intermediate compounds by the same methods employed in this study. In these cases, however, the engineered strains usually produced very small amounts of or no metabolites [29]. We do not know why engineered fungi and actinobacteria show these different phenotypes, but they might be caused by differences in transcription machinery between prokaryotes and eukaryotes. In prokaryotes, including actinobacteria, several genes are usually transcribed polycistronically; thus, disruption of a gene might affect transcription/translation of the other genes in an operon even though the disruption is designed to be an in-frame deletion. In contrast, all genes in fungi are transcribed monocistronically, and thereby unaffected by expression of neighboring genes. If these are the reasons, the strategy employed in this study should be generally applicable to all types of compounds produced by fungi.

## Materials and Methods

### General

Sequence analysis of PCR fragments was performed by the dideoxy chain termination method with an automatic DNA sequencer (Li-Cor, model 4000L). Cell disruption was performed with an Ultrasonic Disruptor (TOMY, UD-200). Analysis of the samples during protein purification was performed using SDS-polyacrylamide gel electrophoresis (SDS-PAGE), and the proteins were visualized by Coomassie brilliant blue staining. Protein concentration was determined by the Bradford method with bovine serum albumin as a standard.

### Strain and Plasmids

*P. amygdali* Niigata-2 was used for production of **1** and preparation of genomic DNA and cDNA. Draft genome sequences were previously determined [17] and used to search for **1** biosynthetic genes. Total RNA of the strain was isolated using the TRIzol® reagent (Invitrogen, USA) according to the manufacturer’s protocol. The fragments containing 5′- or 3′-termini of cDNA were obtained using the SMART™ RACE cDNA Amplification Kit (Clontech, USA) and GeneRacer™ Kit (Invitrogen).

### Cloning and *in vitro* Assay of Methyltransferase (Orf8)

After determination of the coding region by the RACE method, the full length of cDNA of *orf8* was amplified with *P. amygdali* 5′-Ready cDNA as the template and a primer set listed in Table S1 by PCR. The *Eco*RI-*Sal*I-digested fragment was inserted into the same site of pMAL-p2X (*N*-terminal MBP-fused, New England Biolabs). Expression and purification conditions for the recombinant enzyme were essentially the same as those described in the manufacturer’s protocols. After a purity of the recombinant enzyme was checked by SDS-PAGE, the enzyme was used for *in vitro* assay.

The assay mixture for methyltransferase contained, in a final volume of 100 µL, 0.5 mM of **9**, or 0.5 mM **10**, 5 mM of *S*-adenosyl-L-methionine (SAM), 50 mM Tris–HCl (pH 7.5), and a suitable amount of methyltransferase. The reaction mixture was incubated at 30°C for 2 h and the products were analyzed by HPLC. Analytical conditions were as follows: Merck Mightisil RP-18GP Aqua column (250 mm × 4.6 mm) (Kanto Chemicals, Japan); mobile phase of acetonitrile in water (0 to 20 min, 30% AcCN; 20 to 30 min, 30 to 40%; 30 to 50 min, 40 to 70%; 50 to 60 min, 70 to 100%); flow rate, 1.0 mL/min; detection, 205 nm. The steady-state kinetic parameters were determined by fitting to the Michaelis-Menten equation. An assay for determination of the kinetic parameters of **10** contained, in a final volume of 100 µL, 50 mM HEPES (pH 7.5), 5 mM SAM, 0.145 mM methyltransferase, and 7.5 µM to 0.25 mM **10**. The mixture was incubated at 30°C for 15 min. When the concentration of **10** was fixed at 0.5 mM, the concentration of SAM was varied from 10 µM to 2.5 mM. Triplicate sets of enzyme assays were performed at each substrate concentration, and the Hanes-Woolf plot was used for estimation of kinetic constants.

### Cloning and *in vitro* Assay of Acetyltransferase-2 (Orf12)

After detemination of the coding region by the RACE method, the full length of cDNA for *orf8* was amplified with *P. amygdali* 5′-Ready cDNA as the template and a primer set listed in Table S1 by PCR. The *Kpn*I-*Pst*I-digested fragment was inserted into the same site of pCold TF vector (TaKaRa, Japan) to make pCold-AT-2. Expression and purification conditions for the recombinant enzyme were essentially the same as those described in the manufacturer’s protocols. After a purity of the recombinant enzyme was checked by SDS-PAGE, the enzyme was used for *in vitro* assay.

The assay mixture for acetyltransferase-2 contained, in a final volume of 100 µL, 50 µM **14**, 1 mM acetyl-coenzyme A, 137 mM NaCl, 2.7 mM KCl, 12.4 mM phosphate buffer (pH 7.4), and a suitable amount of purified enzyme. The mixture was incubated at 30°C for overnight and the reaction product was analyzed by HPLC. Analytical conditions were same as those of methyltransferase.

The steady-state kinetic parameters were determined by fitting to the Michaelis-Menten equation. An assay for determination of the kinetic parameters of **14** contained, in a final volume of 100 µL, 50 mM Tris–HCl (pH 8.5), 5.06 mM acetyl-coenzyme A, 7.68 µM acetyltransferase, and 50.9 µM to 1.02 mM **14**. The mixture was incubated at 35°C for 30 min. When the concentration of **14** was fixed at 203 µM, the concentration of acetyl-coenzyme A was varied from 23.1 µM to 1.16 mM. Triplicate sets of enzyme assays were performed at each substrate concentration, and the Hanes-Woolf plot was used for estimation of kinetic constants.

### Cloning and *in vitro* Assay of Glycosyltransferase (Orf6)

After determination of the coding region by the RACE method, the full length of cDNA for *orf6* was amplified with *P. amygdali* 5′-Ready cDNA as the template and a primer set listed in Table S1 by PCR. The *Nde*I-*Bam*HI-digested fragment was inserted into the same site of pET15b (Merck, Germany) to make pET15-GLY. The *E. coli* cells having pET15-GLY was grown in LB medium supplemented 100 µg/ml ampicillin. The culture was grown at 37°C until OD_600_ reached 0.5. After addition of 0.5 mM of isopropyl β-D-thiogalactopyranoside, the cultivation was continued for additional 18 hours at 25°C. His-tagged enzyme was purified by using the manufacturer’s protocol. After a purity of the recombinant enzyme was checked by SDS-PAGE, the enzyme was used for *in vitro* assay.

The assay mixture for glycosyltransferase contained, in a final volume of 100 µL, 0.5 mM of **9**, 2.5 mM of UDP-glucose, 50 mM Tris–HCl (pH 7.5), and a suitable amount of glycosyltransferase. The mixture was incubated at 30°C for 2 h and the products were analyzed by HPLC. Analytical conditions were same as those of methyltransferase. The steady-state kinetic parameters were determined by fitting to the Michaelis-Menten equation. An assay for determination of the kinetic parameters of **9** contained, in a final volume of 100 µL, 50 mM MES (pH 6.0), 5 mM UDP-glucose, 0.58 mM glycosyltransferase, and 5 µM to 100 µM **9**. The mixture was incubated at 30°C for 15 min. When the concentration of **9** was fixed at 0.1 mM, the concentration of UDP-glucose was varied from 25 µM to 2.5 mM. Triplicate sets of enzyme assays were performed at each substrate concentration, and the Hanes-Woolf plot was used for estimation of kinetic constants.

### Cloning and Characterization of P450-2 (Orf5)

We examined the function of cytochrome P450-2 (*orf5*) by a heterologous expression in yeast, the detailed procedure of which was described previously [21]. The coding region of the P450-2 was determined using the RACE (Rapid Amplification of cDNA End) method. Then, approximately 1.6 kb of full length DNA fragment was amplified with *P. amygdali* 5′-Ready cDNA as the template and a primer set listed in Table S1. The *Bam*HI-*Xho*I-digested fragment was inserted into pESC-URA to yield pESC-URA-P450-2. For functional analyses of cytochrome P450 enzymes, a cytochrome P450 reductase is essential. We identified a cytochrome P450 reductase gene in draft genome database of the FC producer. The 2.1 kb of full length DNA fragment was amplified with *P. amygdali* 5′-Ready cDNA as the template and a primer set listed in Table S1. The *Eco*RI-*Bgl*II-digested fragment was inserted into the same sites of the pESC-URA-P450-2. The constructed plasimd was introduced into *S. cerevisiae* YPH500 (*his^−^*, *leu^−^*, *trp^−^*, *ura^−^, ade^−^ and lys^−^*) together with pESC-TRP-*orf8*-ADH, which was previously constructed and carried an fusicocca-2,10(14)-diene synthase gene [21]. After cultivation, pentane extract of the culture broth was analyzed by GC/MS, which was conducted with QP2010 apparatus (Shimazu), using a DB-1MS capillary column (0.32 mm × 30 m, 0.25 µm film thickness; J&W Scientific). Each sample was injected into the column at 100°C in a split less mode. After a three-min isothermal hold at 100°C, the column temperature was increased to 250°C at a ratio of 16°C/min, followed by a 3-min isothermal hold at 250°C. The flow rate of the helium carrier was 1 mL/min.

### Cloning and *in vitro* Assay of P450-3 (Orf7)

After determination of the coding region by the RACE method, the full length of cDNA for *orf7* was amplified with *P. amygdali* 5′-Ready cDNA as the template and a primer set listed in Table S1 by PCR. The 1.5 kb PCR product was cloned into pGEM-T Easy vector (Promega Corporation, USA) to yield pGEM450-3. The *Spe*I-*Eco*RI-digested fragment from pGEM450-3 was inserted into the same site of pESC-HIS to construct pESC-P450-3. The constructed pESC-P450-3 was introduced into *S. cerevisiae* YPH500 (*his^−^*, *leu^−^*, *trp^−^*, *ura^−^, ade^−^ and lys^−^*). After cultivation of the transformants in the presence of galactose, microsomal fractions were prepared and incubated with fusicocca-1,10(14)-diene-8β,16-diol. After 48 h incubation at 30°C, the products were analyzed by HPLC. Analytical conditions of HPLC were previously reported [1].

### Transformation of *P. amygdali*

Protoplast preparation: *P. amygdali* Niigata-2 was cultivated by the same method as described previously [15]. Mycelia from 50 ml culture were collected by centrifugation (5,000 × g, 10 min), washed with 0.8 M NaCl twice and then incubated in 10 ml of the same medium with yatalase (5 mg/ml, Takara Bio) and lysing enzyme (10 mg/ml, Novozyme) at 30°C for 2 h. Protoplast formation was monitored microscopically. Protoplasts were recovered by filtration with Miracloth (Calbiochem), centrifuged at 1,800 × g and 4°C for 10 min, and washed twice with ice-cold 0.8 M NaCl. They were gently resuspended in 300 µl of STC buffer (1.2 M of Sorbitol, 10 mM of Tris-HCl (pH 7.5), and 10 mM of CaCl_2_) and immediately used for transformation. Transformation was done with 10 µg of plasmid DNA in 10 µl of 10 mM of TE buffer to which a 100-µl suspension of 10^8^ protoplasts was added. After the mixture was left for 10 min on ice, 1 ml of 60% (vol/vol) polyethylene glycol 4000 in a buffer containing Tris-HCl (10 mM, pH 7.5) and CaCl_2_ (10 mM) was added and mixed gently by pipetting. The mixture was appropriately diluted with STC buffer and 100-µl samples were plated on YPSA plate containing 0.1% yeast extract, 0.1% bacto tryptone, 27.7% sucrose, 0.005% hygromycin, and 2% agar. These plates were incubated at 30°C for 20 h and then overlaid with 5 ml of nutrient soft agar (0.8%).

### Gene Disruption

The *orf10* and the *orf13* genes were disrupted by a double-crossover event. To construct the *orf10* and *orf13* gene disruption plasmids, two 3-kb and 2-kb DNA fragments, carrying upstream and downstream regions of the target genes, respectively, were amplified with the primer set listed in Table S1 by PCR. After sequence confirmation, these fragments were inserted into appropriate restriction sites of pGEM-T Easy in the same direction as in the genomic region. A 3.0-kb DNA fragment containing a Hygromycin resistance gene controlled by a TrpC promoter and terminator was amplified by PCR with pSH75 [30] as the template and the primer set listed in Table S1. After sequence confirmation, this 3.0-kb fragment was then inserted between the above-mentioned two fragments. The constructed plasmids were used to transform the **1** producer (Fig. 4A and 5A). Gene disruption of the transformant was confirmed by PCR analysis (Fig. 4C and 5C) and the PCR product was confirmed by DNA sequencing.

### Structural Analysis of the Reaction Product

The reaction products formed by *in vitro* assays were subjected to LC-MS analysis. The analytical conditions were as follows: Develosil RPAQUEOUS-AR-5 column (150×2.0 mm); column temperature, 30°C; detection, 250 nm and positive mode; mobile phase, 0.1% formic acid:acetonitrile = 10:90 at 0 min, and a linear gradient to 50:50 for an additional 30 min; flow rate, 0.3 ml/min.

## Supporting Information

Figure S1 (A) HPLC analyses of the product formed by *in vitro* methyltransferase assay using FC H aglycon (**9**) (upper), FC H (**10**) (middle), and a mixture of **10** and 3-*epi*-**10** (lower) as substrates. (B) The reaction products formed from **9** (peak A) and **10** (peak B) were confirmed to be 16-*O*-methyl-**9** and 16-*O*-methyl-**10** by LC/MS analysis, respectively.
(DOC)

Figure S2 HPLC (A) and LC/MS (B) analyses of the product formed by *in vitro* acetyltransferase assay. The reaction product formed from dideacetyl-FC A (**14**) was confirmed to be **15** by LC/MS analysis.
(DOC)

Figure S3 HPLC (A) and LC/MS (B) analyses of the product formed by *in vitro* glycosyltransferase assay. The reaction product formed from FC H aglycon (**9**) was confirmed to be **10** by LC/MS analysis.
(DOC)

Figure S4 GC-MS analyses of pentane extract of the broth of *S. cerevisiae* transformant carrying fusicocca-2,10(14)-diene synthase gene, cytochrome P450 reductase gene, and P450-2 gene. Chromatograms were recorded in the TIC mode (A and B). (A) Authentic fusicocca-2,10(14)-diene-8β-ol (**5**). (B) Extract of the transformant. (C) Mass spectrum of authentic **5**. (D) Mass spectrum of the transformant.
(DOC)

Figure S5 HPLC (A) and LC/MS (B) analyses of the product formed by *in vitro* P450-3 assay. The reaction product formed from fusicocca-1,10(14)-diene-8β,16-diol (**8**) was confirmed to be **9** by LC/MS analysis.
(DOC)

Table S1 Primers used for PCR.
(DOC)
