# Supplementary figures and images for: Molecular Breeding of a Fungus Producing a Precursor Diterpene Suitable for Semi-Synthesis by Dissection of the Biosynthetic Machinery
Source: PLoS One. 2012 Aug 1;7(8):e42090. doi: 10.1371/journal.pone.0042090 (PMC3411640; doi:10.1371/journal.pone.0042090)

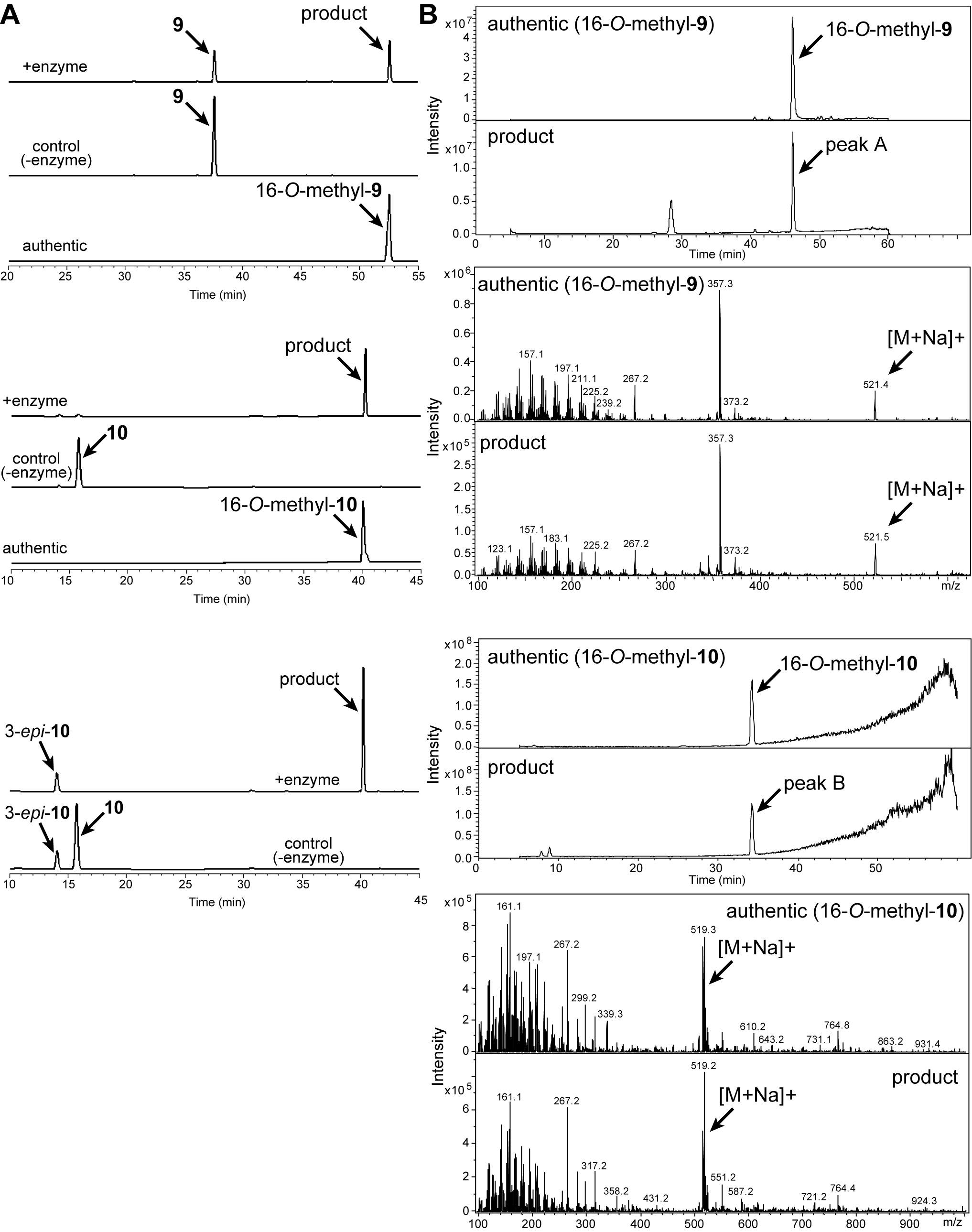


**Figure** **S1.**

Supplement: Figure S1 — (A) HPLC analyses of the product formed by in vitro methyltransferase assay using FC H aglycon (9) (upper), FC H (10) (middle), and a mixture of 10 and 3-epi-10 (lower) as substrates. (B) The reaction products formed from 9 (peak A) and 10 (peak B) were confirmed to be 16-O-methyl-9 and 16-O-methyl-10 by LC/MS analysis, respectively. (DOC) [file pone.0042090.s001.doc]

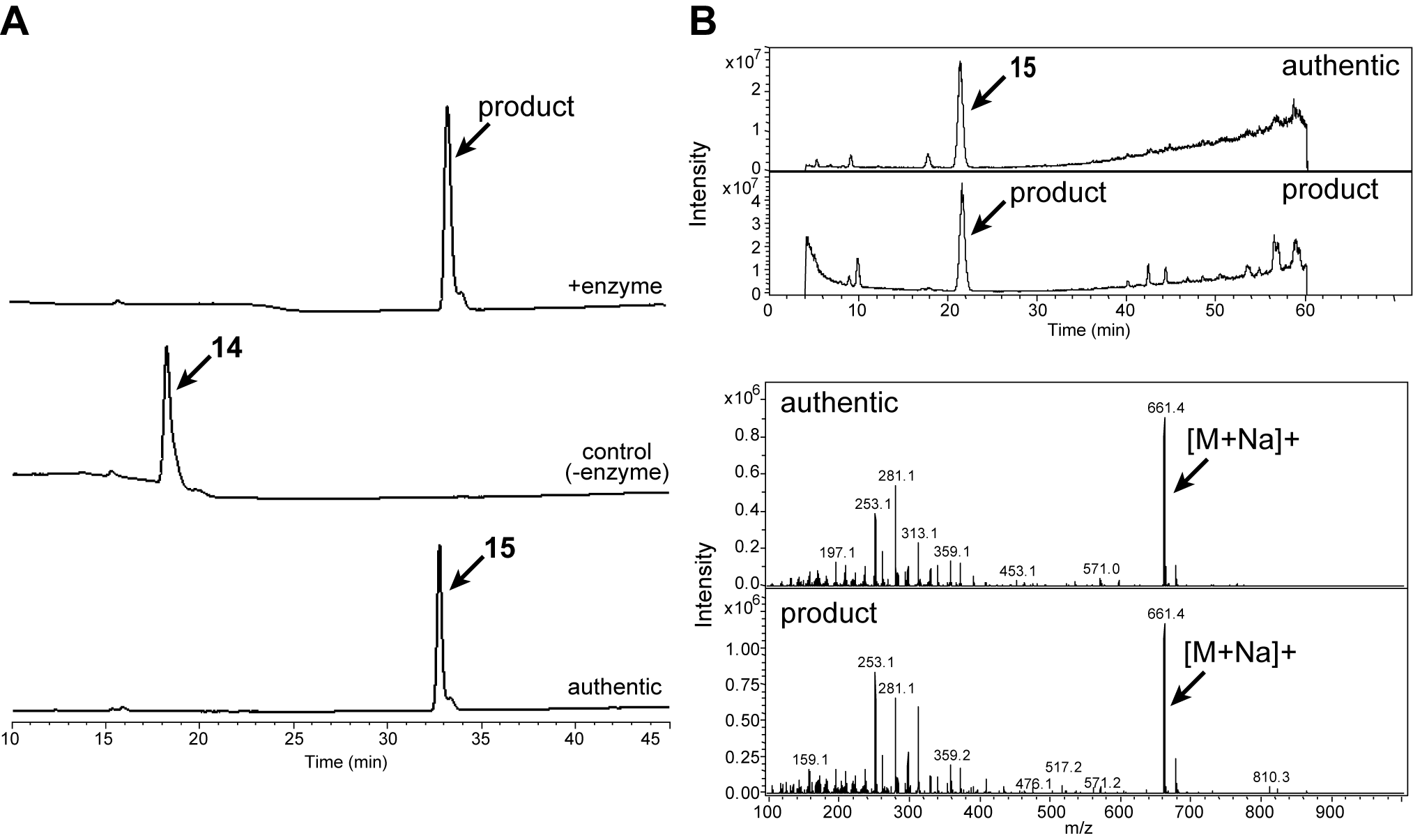


**Figure** **S2.**

Supplement: Figure S2 — HPLC (A) and LC/MS (B) analyses of the product formed by in vitro acetyltransferase assay. The reaction product formed from dideacetyl-FC A (14) was confirmed to be 15 by LC/MS analysis. (DOC) [file pone.0042090.s002.doc]

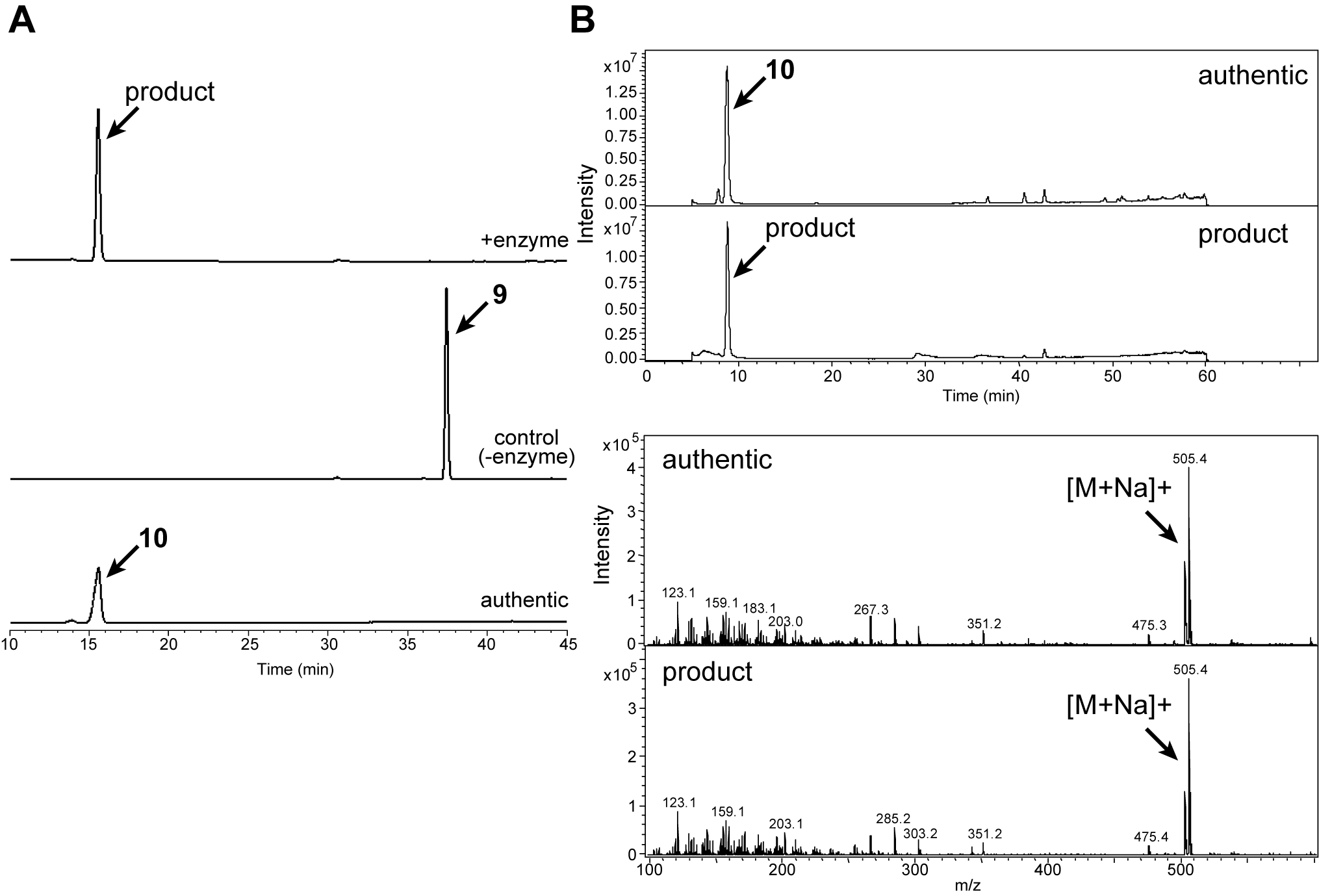


**Figure** **S3.**

Supplement: Figure S3 — HPLC (A) and LC/MS (B) analyses of the product formed by in vitro glycosyltransferase assay. The reaction product formed from FC H aglycon (9) was confirmed to be 10 by LC/MS analysis. (DOC) [file pone.0042090.s003.doc]

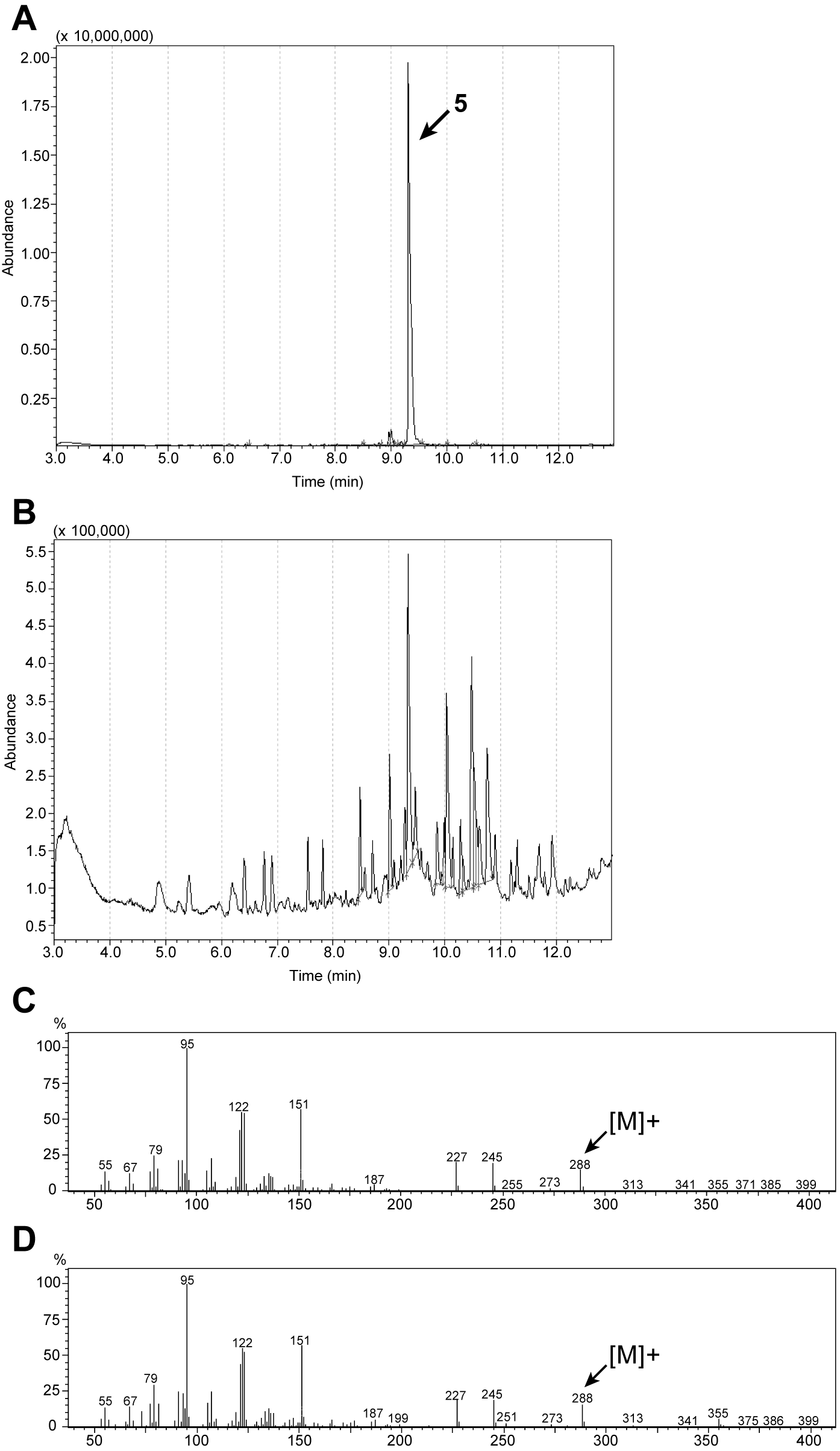


**Figure** **S4.**

Supplement: Figure S4 — GC-MS analyses of pentane extract of the broth of S. cerevisiae transformant carrying fusicocca-2,10(14)-diene synthase gene, cytochrome P450 reductase gene, and P450-2 gene. Chromatograms were recorded in the TIC mode (A and B). (A) Authentic fusicocca-2,10(14)-diene-8β-ol (5). (B) Extract of the transformant. (C) Mass spectrum of authentic 5. (D) Mass spectrum of the transformant. (DOC) [file pone.0042090.s004.doc]

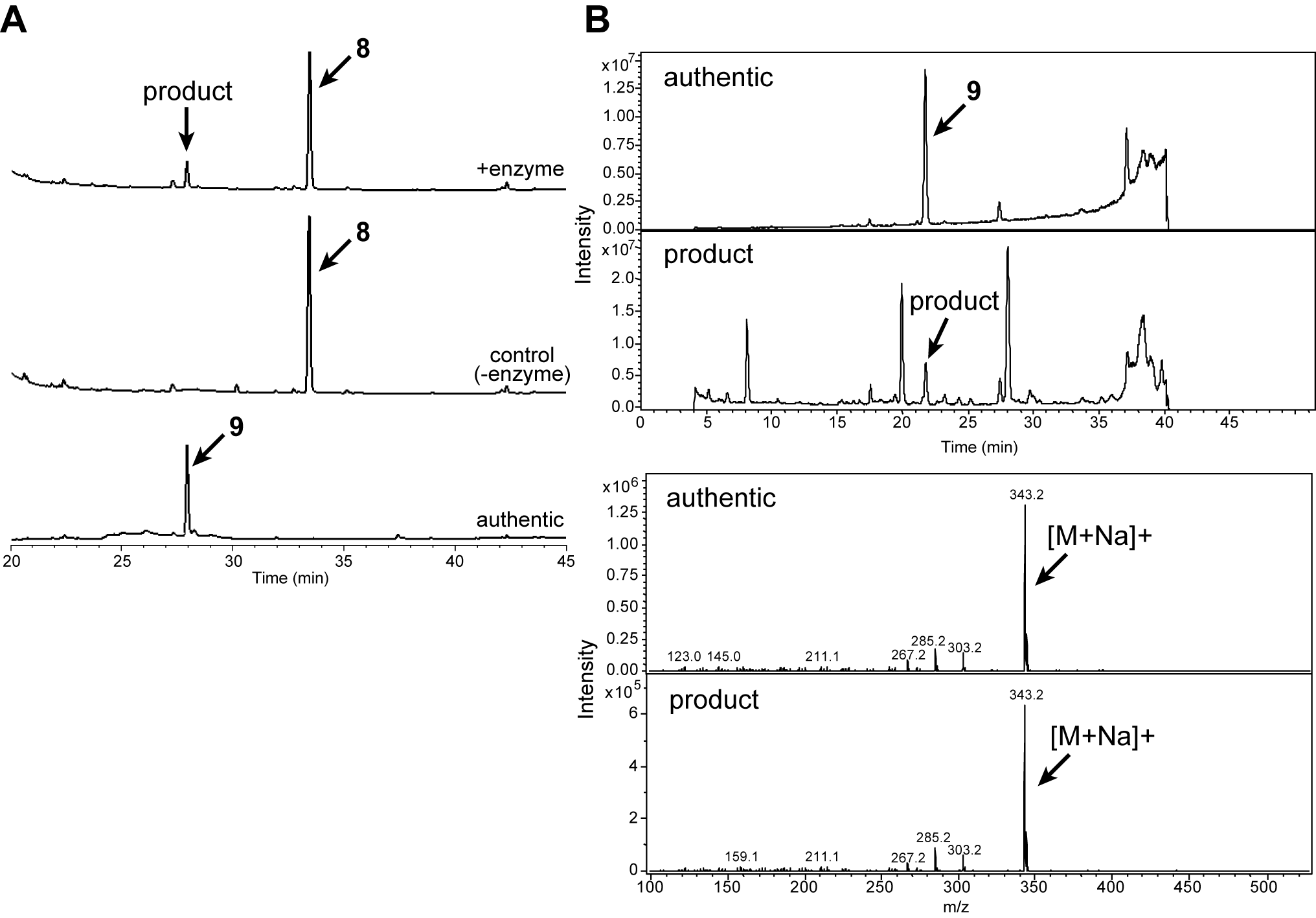


**Figure** **S5.**

Supplement: Figure S5 — HPLC (A) and LC/MS (B) analyses of the product formed by in vitro P450-3 assay. The reaction product formed from fusicocca-1,10(14)-diene-8β,16-diol (8) was confirmed to be 9 by LC/MS analysis. (DOC) [file pone.0042090.s005.doc]
