# Supplementary material for: Molecular Breeding of a Fungus Producing a Precursor Diterpene Suitable for Semi-Synthesis by Dissection of the Biosynthetic Machinery
Source: PLoS One. 2012 Aug 1;7(8):e42090. doi: 10.1371/journal.pone.0042090 (PMC3411640; doi:10.1371/journal.pone.0042090)
Supplement: Table S1 — Primers used for PCR. (DOC) [file pone.0042090.s006.doc]

**Table S1.**

| Primer sequences used for PCR (5’ to 3’) | Note |
| --- | --- |
|  |  |
| CCGGAATTCGGATCCCATGGCTGAACTCGACACTCTG | Used for amplification of |
| CCGAATTCGTCGACCTATGACCAGACATCCTCCTG | cDNA of P450-2 (*orf5*) |
|  |  |
| AGTACTTTGACTCATATGTCCCCGCCTTCACAAATCAAACC | Used for amplification of |
| CTGCAGAGGATCCTCAATTGGTGGAAGGAAATGGTTC | cDNA of glycosyltransferase (*orf6*) |
|  |  |
| ATGCTCTCCACCATGGACACCGTGG | Used for amplification of |
| TCAACCTGGTAACTTAACTTCCTCTGC | cDNA of P450-3 (*orf7*) |
|  |  |
| ATACTGTGAATTCATGGATGACAGCAAGACAAACGGCC | Used for amplification of |
| TGCAGTCGTCGACTTAGGCGTTGAAGACTACTTCAATC | cDNA of methyltransferase (*orf8*) |
|  |  |
| GGCGGTACCGGCATGTCTCAAACAACCGTCCCTGTC | Used for amplification of |
| CCGCTGCAGGCCTCAAGGCAGCTTTTGCATCTCC | cDNA of acetyltransferase-2 (*orf12*) |
|  |  |
| CCGGAATTCCGATGGCTGAACTCGACACTCTG | Used for amplification of |
| CCGGAATTCAGATCTCTATGACCAGACATCCTCCTG | cDNA of cytochrome P450 reductase |
|  |  |
| GCATGCATGGCGGCACGCAGGAGCCGCTTG | Used for amplification of |
| GCATGCGAAGACTGCCGCGCACTATGGTAG | an upstream region of *orf10* |
|  |  |
| GCGGCCGCCTCTGAGTGCCTTCAGATAACAATTTC | Used for amplification of |
| GCGGCCGCGCAAGCTCATCTACCTCCCGGCTAG | a downstream region of *orf10* |
|  |  |
| GCGGCCGCGATCGACGCACCGTATGTGC | Used for amplification of |
| GCGGCCGCGTGACCTTCAAATGGTGCGT | an upstream region of *orf13* |
|  |  |
| GCATGCCAGTGCCGTTCAACAGGGGC | Used for amplification of |
| GCATGCAGACACGCCGTACTGTACGC | a downstream region of *orf13* |
|  |  |
| ATCAGTATCTCGAGATGCCAGTTGTTCCAGTGAT | Used for amplification of |
| TGCAGAGGCTCGAGAATTAAGTCTAGAAAGAAGG | a hygromycin-resistance gene cassette |
|  |  |
| GACCACCACGCAACTACCATAGTGCGCG | Used for confirmation of |
| CTCCAGTGAAATTGTTATCTGAAGGCAC | *orf10* disruptant |
|  |  |
| GAATATACATCTGAACTTGACGGTAACG | Used for confirmation of |
| CTTAACCGTTCCGGAGCCCCTGTTGAAC | *orf13* disruptant |
|  |  |
